# Machinery for potato harvesting: a state-of-the-art review

**DOI:** 10.3389/fpls.2023.1156734

**Published:** 2023-05-22

**Authors:** Ciaran Miceal Johnson, Fernando Auat Cheein

**Affiliations:** UK National Robotarium, School of Engineering and Physical Sciences, Heriot-Watt University, Edinburgh, United Kingdom

**Keywords:** potato harvesting, automation, machinery, internet of things, artificial intelligence, robotics

## Abstract

Potatoes are the fourth most important crop for human consumption. In the 18 century, potatoes saved the European population from starvation, and since then, it has become one of the primary crops cultivated in countries such as Spain, France, Germany, Ukraine and the United Kingdom. Potato production worldwide reached 368.8 million tonnes in 2019, 371.1 million tonnes in 2020, and 376.1 million tonnes in 2021, with production expected to grow alongside the worldwide population. However, the agricultural sector is currently suffering from urbanization. With the next generation of farmers relocating to cities, there is a diminishing and ageing agricultural workforce. Consequently, farms urgently need innovation, particularly from a technology perspective. As a result, this work is focused on reviewing the worldwide developments in potato harvesting, with an emphasis on mechatronics, the use of intelligent systems and the opportunities that arise from applications utilising the Internet of Things (IoT). Our work covers worldwide scientific publications in the last five years, sustained by public data made available from different governments. We end our review by providing a discussion on the future trends derived from our analysis.

## 1 Introduction

Around the world, the strain placed upon agriculture is compounding. A diminishing pool of skilled laborers, the impact of climate change, and an ever-increasing human population are a few of the challenges facing modern agriculture. Potatoes, as the fourth most grown crop in the world behind wheat, rice, and corn, will play a large role in feeding the increasing population [Zhang et al. (2017); Jennings et al. (2020); Issa et al. (2020)]. Ensuring an efficient potato production pipeline is of great importance. The stage of the potato production pipeline which suffers the greatest losses is harvesting [Spang and Stevens (2018)]. Potato harvesting is the process of separating and collecting potato tubers from the soil. During this, losses occur as potatoes are damaged or left in the field.

**Figure 1:**
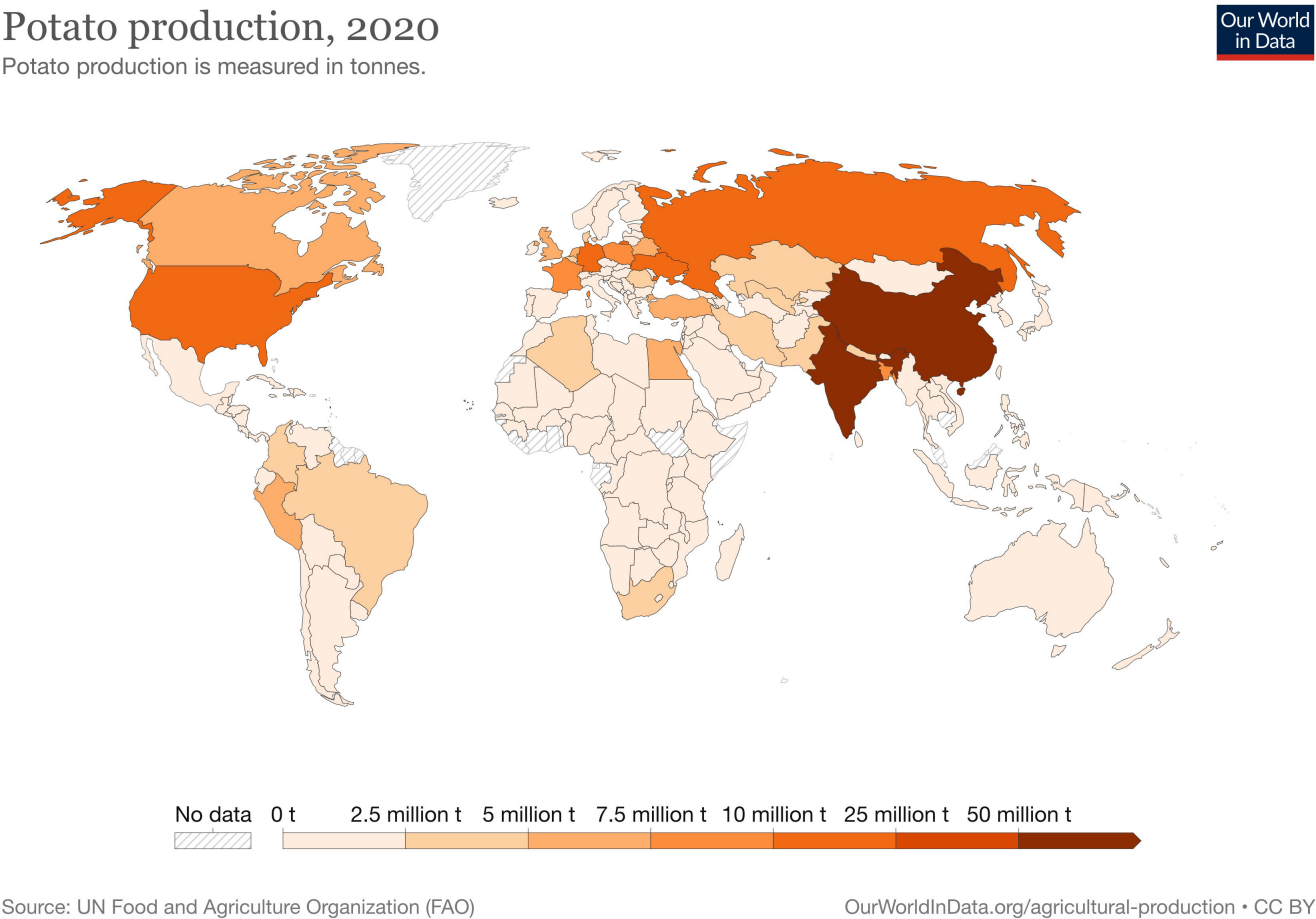
Heatmap of the global production of potatoes in 2020 (in millions of tonnes). Image taken from Ritchie et al. (2023) using data made available by Dataset FAO (2022b).

There is not a single potato harvesting solution which generalizes well to all farms, geographies, and soil types. The mechanical design of potato harvesters depends heavily on the environment in which it operates. Regional factors along with the available harvesting methods can greatly impact potato production [Wei et al. (2019)], as can be seen in **Figure 1**. The production in the northern and central parts of the globe, which use mechanical harvesting, is significantly higher than in the southern hemisphere. There is also a great variation within hemispheres which is worth exploring in more detail. It is important not to simplify the problem, but to view the geographical and political issues which may arise when proposing certain solutions to potato harvesting.

Potatoes can be harvested using a variety of equipment. The most simplistic method of harvesting is manual. This can be done using a hand hoe, spading fork or even without any equipment. Harvesting by hand is a time-consuming and labor-intensive task [Gulati (2019)]. Therefore animal-drawn harvesters, such as the traditional plough, were deployed to solve these problems. Both methods of harvesting are still common practice in many parts of the world, despite draught animals being neglected and even sometimes harmed. Many veterinarians and animal welfare organizations continually advocate for an improvement in their living and working conditions [Ramaswamy (1998); Mota-Rojas et al. (2020)]. A step up in complexity introduces semi- and fully-mechanised harvesters. The difference is that fully-mechanised harvesters collect the potatoes in a trolley or bunker during harvesting, saving the manual labor required to collect the potatoes from the field by hand after harvesting. Mechanical harvesters are considered an improvement on the first two methods of harvesting as they reduce harvesting time, cost, and losses [Nasr et al. (2019); Soethoudt and Castelein (2021)]. Finally, there has been discussion regarding the automation of potato harvesters, though there is no working prototype in academic literature or at an industrial scale implementation [McPhee et al. (2020)].

This review will begin by looking into the current state of global potato harvesting, diving into the geographical differences and discussing reasons for these differences. Followed by potato harvesting constraints which may impact harvesting. These are potato and soil characteristics. The technology used in potato harvesting will be reviewed, starting with the mechanical harvester specifications and design. Followed by the future trends of potato harvesting. Finally, a discussion will be provided on the state of potato harvesting around the world, with the goal of specifying an automation level for the top-producing potato countries in each continent.

This work only considers scientific journal articles released from January 2017 – December 2022, and information available from governmental agencies. For a fair analysis, we kept our emphasis on articles from countries with available agricultural information. The selected articles were obtained through the Scopus database. Articles under the subject areas of Chemistry; Medicine; and Biochemistry, Genetics and Molecular Biology were automatically filtered out from the search. We also restricted the articles to only those with an English version. The focus on the selection of articles was put on the machinery for potato harvesting.

## 2 Potato harvesting: an international assessment

Potato harvesting is complex, with various different factors preventing farmers and scientists from finding an optimum –and unique– harvesting solution. The geographical location for example can impact the optimum harvesting solution due to variations in terrain, climate and soil characteristics. Consequently, farmers around the world require bespoke solutions to harvesting. The societal role of potatoes around the world also varies. The majority of potato farms in Asia, South America, and Africa are smallholders [Devaux et al. (2021)]. They treat potatoes as a staple crop and not necessarily as a cash crop. A staple crop is used to feed the general population and constitutes a significant proportion of the nation’s diet. Cash crops on the other hand are grown in order to generate profit. There is a drive for these smallholders to increase their productivity by utilising modern farming techniques [Devaux et al. (2021); Wu et al. (2018)]. However, such techniques must be tailored to the farm in which they are deployed.

### 2.1 Potato production by continent and country

**Figure 2:**
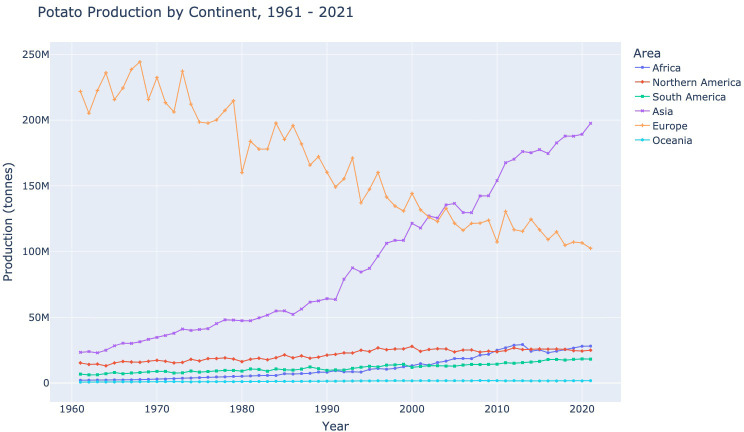
Potato production by continent for the years 1961 - 2021 (in millions of tonnes), using data made available by Dataset FAO (2022b).

The worldwide potato production landscape has changed in recent years, as shown in **Figure 2**. Formerly the highest potato-producing continent, Europe has experienced a large decline in potato production being surpassed by Asia as the top-producing continent. Africa also shows a rapid increase in potato production, while Oceania, South and North America display steadier growth.

**Figure 3:**
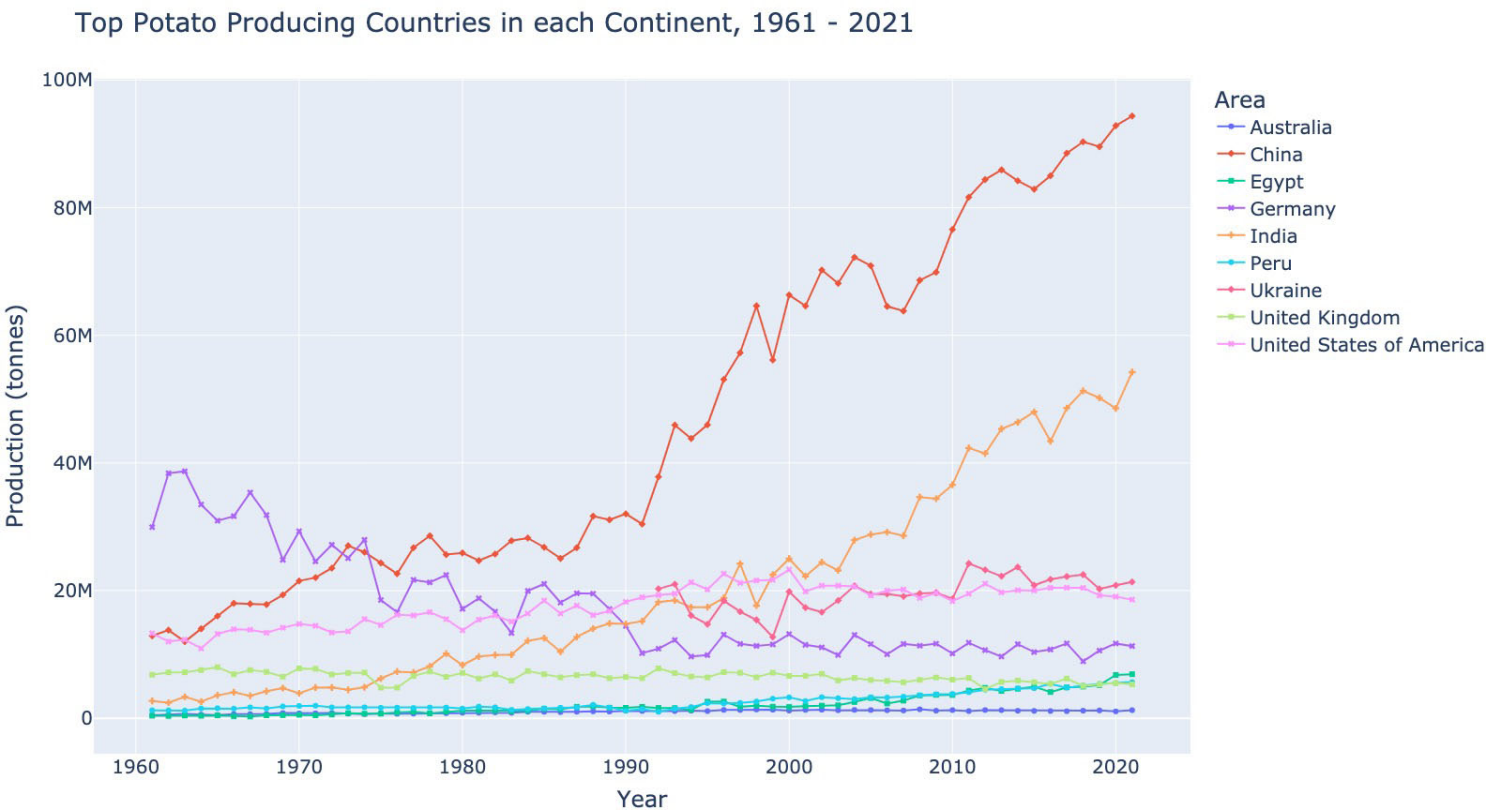
Top potato-producing countries by continent, including India, Germany, and the United Kingdom for the years 1961 - 2021 (in millions of tonnes), using data made available by Dataset FAO (2022b).

The top potato-producing countries in each continent will be studied in this section. These are China, Ukraine, the USA, Peru, and Australia. Another country included in this section is India, as they are the second largest potato producer in the world after China. Germany, as they are the largest Western European potato producer. And the UK, as they recently left the European Union. The potato production, in tonnes, for each of these countries from 1961–2021 can be found in **Figure 3**.

An in-depth study of these countries will be provided for the years 2017–2021 since this review only considers scientific journal articles released from 2017–2022. Data for 2022 is not provided as it was not made available at the time of this review.

Potato production provides a one-dimensional view of a country’s ability to grow and harvest potatoes. Larger countries can dedicate more land to growing and ultimately will produce more potatoes. This does not mean that they are efficient with their land use. In order to provide an insight into their efficiency we look at yield. Yield is the quantity of potatoes produced in a given area. Finally, the population of a country is discussed. A higher population may result in a greater need to produce potatoes in order to feed their population. Though a high population may also restrict their land use.

### 2.2 Asia

China and India are the top potato-producing countries in the world. Since China achieved the top spot in 1993, the nation has been pushing campaigns to increase its consumption of this food group [Devaux et al. (2021)]. Harvesting in China is split between fully- and semi-mechanized harvesters, with the majority of harvesting being semi-mechanized [Wei et al. (2019); Issa et al. (2020); Fu et al. (2022)]. Due to the heavy clay soil found in Northern China, their research revolves around removing soil after extraction [Fu et al. (2022); Wei et al. (2019)]. Currently, soil clods and stones are removed manually after harvesting. Though China is doing research into the use of computer vision to automate their removal (see Fu et al. (2022) and the references therein). India is also primarily a semi-mechanized harvesting nation [Gulati (2019)]. Though they are moving towards fully-mechanised harvesters, such as the one proposed by Gulati (2019).

**Figure 4:**
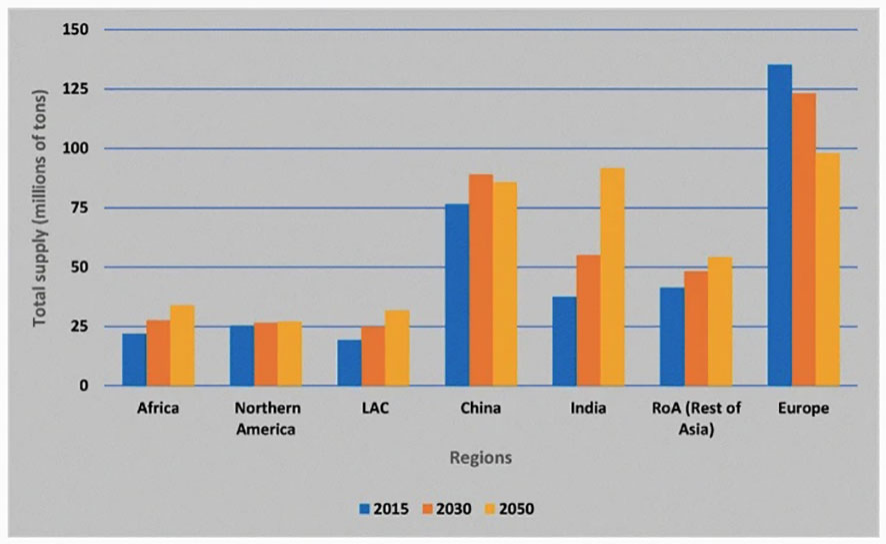
Prediction of future potato production taken from Devaux et al. (2021) adapted from Rosegrant et al. (2017).

By 2050, Rosegrant et al. (2017) predicts that China will be surpassed by India as the top potato-producing country. The results found by Rosegrant et al. (2017) was adapted by Devaux et al. (2021) producing the bar chart seen in **Figure 4**. Currently, India ranks second in potato production, population and area harvested. Although India has a higher yield than China it is still far smaller than other countries included in the survey. It is unclear whether improving yield will lead to higher production, as the reduction in yield may be due to factors such as continuous monoculture growing. Continuous monoculture growing can lead to disease 107 which reduces yield however continually growing potatoes may be the reason for higher production.

### 2.3 Western Europe

Before Brexit, 60% of the European (EU-28) potato production was produced in five Northwestern European countries. These countries are referred to in Goffart et al. (2022) and Devaux et al. (2021) as the NWEC-05. The NWEC-05 is made up of Germany, Belgium, France, Netherlands, and the UK. It is worth mentioning that the UK is no longer a member of the European Union, and therefore will be discussed separately.

The high level of mechanization seen in NWEC-05 is expensive [Goffart et al. (2022)]. Such costs are justified as these are advanced and profitable sectors for the countries. This high level of automation in industrial farms is partially due to the fact that potatoes are seen as cash crops as well as staple crops in these countries. A high proportion of the crops are sold to processing companies. For example, in Belgium, only 20% of the potatoes are sold as fresh produce while the remaining 80% are sold for processing [Devaux et al. (2021)].

The top potato-producing country in Western Europe is Germany. As can be seen in **Figure 2**, potato production in Europe is declining. This is evident in the data provided by Germany. Between 2017–2021, a slight decline in production and an increase in the harvested area saw a large reduction in Germany’s yield. Germany also had the smallest variation in population across the five years.

#### 2.3.1 United Kingdom

As a member of NWEC-05, the UK was one of the top-producing potato countries in Europe. Similar to Germany, it has experienced a reduction in yield between 2017–2021. A noticeable difference however is that while Germany produced slightly fewer potatoes (-3.5%) by using more land (+3.1%); the UK produced significantly fewer potatoes (-14.7%) while using less land (-6.2%).

**Figure 5:**
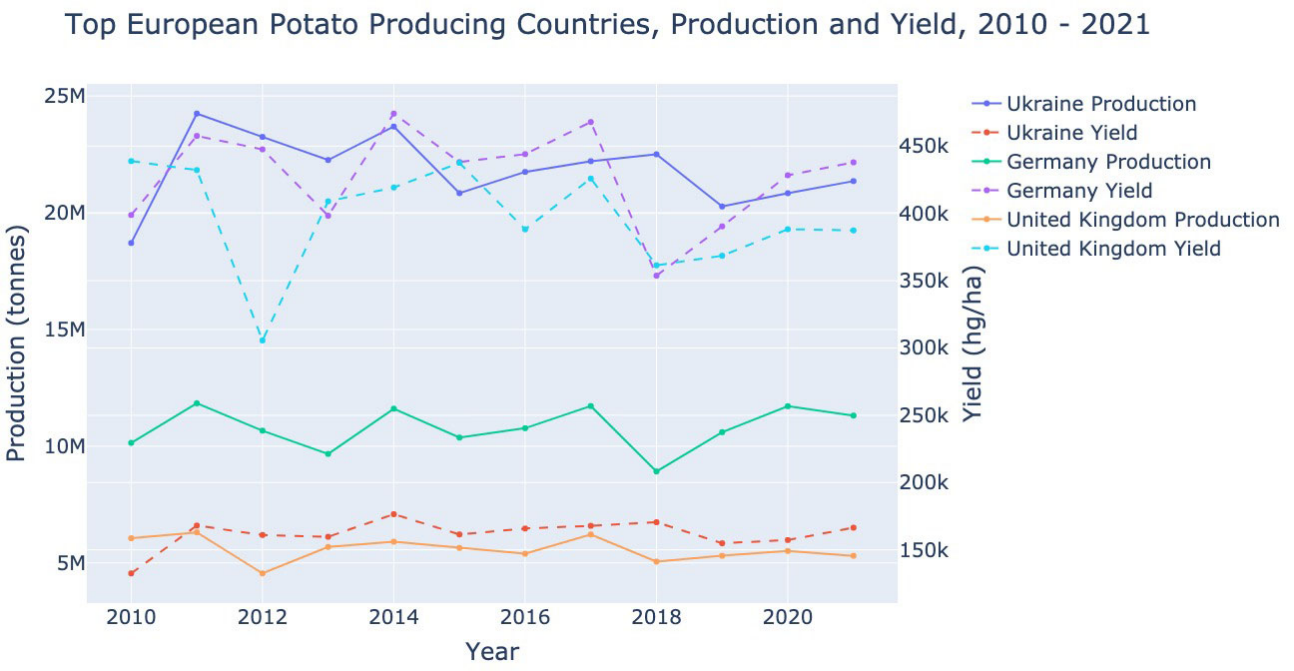
Potato production (in tonnes) and yield (in hg/ha) for Germany, Ukraine and the United Kingdom from 2010–2021. Data extracted from Dataset FAO (2022b).

**Figure 5**, shows the potato production and yield for the three European countries discussed in this review: Germany, the UK, and Ukraine. Both Germany and the UK experience a local maximum in 2017 followed by a steep reduction in production and yield. These values begin to recover towards 2021 with Germany’s recovering more quickly. This data shows that production can be greatly disrupted in one year and it may take several years to recover.

### 2.4 Eastern Europe

The third largest potato-producing country in the world behind China and India is Ukraine. Ukraine is a very active member of the potato harvesting research community. They are a fully-mechanised industry, although a significant number of the machines used to grow potatoes are imported from Russia, Belarus, and Germany [Hrushetsky et al. (2019); Hrushetskyi et al. (2021)]. Due to their heavy loam soil, the majority of research papers discuss the removal of soil clods from the harvesting process [Bulgakov et al. (2017; Bulgakov et al., 2019; Bulgakov et al., 2020; Bulgakov et al., 2021)]. The harvesting may be fully-mechanised however the removal of soil clods is still done manually which can be labor-intensive and expensive [Bulgakov et al. (2021)].

Referring back to **Figure 5**, it clearly shows that Ukraine produces more potatoes than its European counterparts, with drastically lower yet more stable yields. These low yields may be indicative of the loss found when harvesting in the heavy loam soil. Ukraine, like the UK, experienced a decrease in potato production, yield, and the harvested area between 2017–2021. However; Ukraine alone experienced a steady reduction in population between 2017–2021.

### 2.5 North America

In North America, like NWEC-05, potatoes are treated as cash crops: with US potato production in 2021 equating to 410 million cwt and processing accounting for 281 million cwt [USDA (2022)]. Farmers can optimize their financial returns by meeting certain incentives in their contracts with processing companies [Waxman et al. (2018)]. Mechanical approaches to harvesting can help the farmer meet these incentives.

The United States of America experienced the highest yield of any country in the survey and even managed to increase their yield by +1.4% between 2017–2021. They experienced a decrease in production (-9.2%) however since their harvested area decreased by a large amount (-10.4%), their yield was not negatively affected. They also had an increase in population, which is the third largest population in the world behind China and India. However, unlike the other two, their potato production ranking does not equate to their population ranking.

### 2.6 South America

In countries such as Argentina, Brazil and Peru, potatoes are harvested mainly by semi-mechanised methods. In Argentina for example, only 10% of fresh potatoes are harvested by fully-mechanised approaches [The Bureau of the Netherlands Agricultural Council in Buenos Aires (2008)]. Fully-mechanised approaches are more common in processed potato production, these are also often performed on larger areas of land. Semi-mechanised potato harvesters extract the potato from the soil and leave them in rows on top of the soil. The potatoes are then collected by hand and stored in large bags. These bags can remain in the field for up to 12 weeks, which ultimately results in large losses. In The Bureau of the Netherlands Agricultural Council in Buenos Aires (2008), it is suggested that harvesting can be performed better in fresh potato production systems with a mechanical method of picking up, cleaning, grading and bagging the potatoes after extraction from the soil.

Peru is the largest potato-producing country in South America. They experienced the greatest percentage increase in yield (+11.3%) while also having the smallest variation in yield across all countries in the survey. Peru also greatly increased its production (+18.5%) and harvested area (+6.5%) over the five years. Their population almost grew by the largest percentage between 2017–2021, just behind that of Egypt.

### 2.7 Africa

The papers discussing the agricultural landscape of Egypt and Eritrea state that it is constituted of many smaller farms [Nasr et al. (2018); Ghebreagziabiher et al. (2022)]. Such smallholder farmers will likely require smaller harvesters. This is the exact problem addressed in Nasr et al. (2018), where they proposed a semi-mechanised potato harvester for smallholder farms. Africa has the potential to increase potato production in the next few years through input intensification rather than area expansion, due to the increasing population [Devaux et al. (2021)]. Increasing potato production without increasing the area means an improvement in yield. This is beneficial since Sub-Saharan Africa suffers from a yield gap [Harahagazwe et al. (2018)].

Egypt experienced the highest percentage increase in production (+42.6%), population (+50.7%), and harvested area (+7.3%) between 2017–2021. Despite their yield decreasing by -5.4% during this time period, it remained higher than that of China, India, Ukraine and Peru showing that Egypt does not suffer the same yield gap as that seen in Sub-Saharan Africa.

### 2.8 Oceania

The top potato-producing country in the Oceanic continent is Australia. Potatoes are of great importance to Western Australia, as behind wine it is their second highest value-adding horticultural industry and their second highest value vegetable crop behind carrots [Dataset Government of Western Australia, A (2018)]. Nevertheless, compared globally, the country’s production is low. Recent research conducted in Australia proposed the use of a fleet of small to medium-sized fully-autonomous potato harvesters [McPhee et al. (2020)]. Although this proposal displayed the highest level of automation out of all papers considered for this review, it was never implemented.

Australia experienced the smallest variation in production and area harvested during 2017–2021. Along with India and Peru, it is one of the only countries to experience a percentage increase in all four metrics between 2017–2021. Additionally, Australia had the smallest average production, harvested area and population of any country in the study.

## 3 Potato harvesting constraints

The efficiency of the potato harvesting process is affected by a number of issues. These range from environmental issues to farm management practices. This section will be focused on two specific issues, those exclusively related to the plant and those related to soil characteristics.

### 3.1 The potato characteristics that impact harvesting

Understanding the characteristics of different potatoes can result in better designed harvesters. Consideration of such characteristics during the design of mechanical harvesters and post-harvesting hardware can increase yield and reduce waste. For example, Ahangarnezhad et al. (2019) studied the *Agria* variety of potato and split the potato characteristics into physical and mechanical properties. The physical properties include the geometric and arithmetic mean diameter, which is important when designing potato sorting and packaging machines in order to reduce losses during transportation. The mass and volume of the potatoes are also physical properties, which should be considered when designing mechanisms for separating potatoes from other materials during harvesting.

However, when reducing waste, Ahangarnezhad et al. (2019) considers mechanical properties as the fundamental information required to design harvesting or post-harvesting machinery. Mechanical properties include the elasticity module, deformation energy, and fracture force. These properties can be determined by a uniaxial compression test. This test can generate a force-deformation graph, which plots the impact force against the penetration depth. When plotting the compression and restitution within the same graph, the area under the graph represents the energy absorbed by the potato. The energy absorbed by the potato is relevant as high energy absorption equates to high bruise damage [Surdilovic et al. (2018)].

It is to be noted that Ahangarnezhad et al. (2019) showed that many physical properties such as length, width, mass, and geometric mean diameter had a direct relationship to the potato size, while density had an inverse relationship. Relative density, also known as specific gravity, is one of the most important indicators of potato quality (see Waxman et al. (2018) for further reading). This is an estimate of the dry matter content of the potato, providing an indication of its water content. The water content of potatoes is relevant since, as stated by Surdilovic et al. (2018), potatoes with a higher water content experience less force yet higher deformations. Since higher levels of deformation equates to higher potato damage, possessing a high specific gravity is a desirable characteristic. This allows harvesters to move faster and exert more force on the potatoes while maintaining the same level of damage.

The specific gravity of potatoes can be influenced by a variety of factors. For example, Waxman et al. (2018) showed that the specific gravity can be influenced by harvest time and species of potato. Three potato varieties (Russet Burbank, Clearwater Russet, and Alpine Russet) were grown with harvest timings standardized based on that of Russet Burbank, a popular variety of potato used in the processing industry. There were three harvest timings used: approximately 2 weeks prior to normal harvest (early), normal Russet Burbank harvest time (normal), and approximately 2 weeks past normal harvest (late). They determined the specific gravity of the potatoes by two methods, weight-in-air and weight-in-water. A low specific gravity was indicative of an early harvest and a declining specific gravity was that of a late harvest. They also found that the species of potato had an impact on the specific gravity. Clearwater Russet exhibited the highest specific gravity in both years of the experiment.

Potatoes can be bred to have desirable characteristics such as a higher specific gravity. In Melito et al. (2017), an evaluation index is proposed to support the selection of clones with interesting trait combinations. As a result, they compared the tuber yield, specific gravity, chipping ability and earliness. They found a 48% higher productivity in clones compared to the best control. The various clone families had significantly different tuber specific gravity, with 70% of clones having a higher score than 1.080 which is the minimum required to be used in the processing industry. Potato processing contracts often contain Incentive Adjusted Prices (IAP) which provide farmers with financial incentives to produce higher quality potatoes. A common criterion in IAPs is producing potatoes over a certain specific gravity. Consequently, potatoes with a higher specific gravity are not only easier to harvest but also financially beneficial to the farmer.

### 3.2 The soil characteristics that impact harvesting

Applying the correct agronomic practices for a potato species can greatly improve the quality of potatoes produced. Agronomic practices and potato characteristics, such as flesh color, can impact the nutrition required to optimally grow and harvest potatoes [Vaitkevičienė et al. (2020)]. Furthermore, throughout the growth cycle, the nutritional demand and therefore availability of nutrients in the soil varies. This temporal availability of nutrients can be utilized by planting multiple species of crops in close proximity. This is called intercropping.

The goal of intercropping systems is to achieve a Land Equivalent Ratio (LER) > 1 [Dong et al. (2018)]. This would suggest that the crops are temporally or spatially cooperating and sharing resources. Conversely, an LER < 1 means the crops are in competition for resources and no benefit is gained from the intercropped system. Intercropping systems have multiple benefits such as reducing weeds and disease. Potato harvester designs should consider that there may be other crops, particularly above-ground crops, in close proximity to the potatoes. Farmers can also get similar benefits from crop rotation [Khakbazan et al. (2019)]. Reducing the load placed upon the farmer by maintaining multiple crops concurrently.

Finally, the soil type and water content can greatly impact tuber damage and loss when harvesting [Bulgakov et al. (2021); Wei et al. (2019)]. Heavy loam soil is considered particularly difficult to harvest as it is prone to compaction. This compaction leads to large soil clods getting extracted with the potatoes which in turn bruise and damage the potatoes. A low water content can also increase the probability of bruising and damaging the potato when harvesting [Wei et al. (2019)]. Soil water content can be controlled through irrigation [Tang et al. (2019)]. Irrigation can ensure that potatoes grow optimally and do not experience water stress. However, this can negatively impact the environment. As a result, the environmental impact should be minimized while also maximizing long-term yield [Tang et al. (2019)]. **Table 1** displays the soil type and water content of the soil in literature. As can be seen in the table, several works discuss the soil type but fewer discuss water content. Reporting these values can help to improve the repeatability of experiments and also help identify trends that arise due to these variables.

**Table 1:** Potato harvesting papers from 2017–2022, the country of their experiment, and soil characteristics that impact harvesting.

Publication	Country	Soil Type	Water Content
Muneer and Dowell (2022)	Scotland	–	–
McPhee et al. (2020)	Australia	Clay loam	–
Issa et al. (2020)	China	Sandy clay	23.8
Fu et al. (2022)	China	Heavy clay	–
Wei et al. (2019)	China	Sandy, clayey	15.6
Tang et al. (2019)	China	–	–
Dong et al. (2018)	China	Orthic anthrosol	–
Bulgakov et al. (2021)	Ukraine	Heavy loam	15–25
Hrushetskyi et al. (2021)	Ukraine	Average loam	16.5
Bulgakov et al. (2017)	Ukraine	Medium loamy	11
Hrushetsky et al. (2019)	Ukraine	Loamy and sandy	–
Bulgakov et al. (2020)	Ukraine	–	11
Bulgakov et al. (2019)	Ukraine	–	–
Poppa et al. (2020)	Germany	–	–
Surdilovic et al. (2018)	Germany	–	–
Schneider et al. (2019)	Austria, Germany	–	–
Nasr et al. (2018)	Egypt	Clay loam	–
Ghebreagziabiher et al. (2022)	Eritrea	–	–
Melito et al. (2017)	Italy	–	–
Sibirev et al. (2019)	Russia	Sandy	21.5
Gulati (2019)	India	Sandy to sandy loam	–
Khakbazan et al. (2019)	Canada	Silty clay loam	–
Vaitkevičienė et al. (2020)	Lithuania	–	–
Vezirov et al. (2021)	Bulgaria	–	–
Ahangarnezhad et al. (2019)	Iran	–	–
Waxman et al. (2018)	USA	Silt loam	–

The soil characteristics are soil type and water content.

- means no data reported.

## 4 Mechanical harvesters

### 4.1 Mechanical harvester specifications

When harvesting potatoes, a common design option is the tunable parameters. These parameters can be adjusted in the field to optimize the performance of the harvester. The characteristics of the potato and the soil can influence the optimal parameters. This review will focus on the forward and conveyor speed of the harvesters as well as the digging depth and angle. Forward speed is the velocity of the harvester as it moves along the farm when harvesting. Conveyor speed is the velocity of the conveyor belt that lifts the potatoes out of the soil and places them in a collection device or in windrows. The digging angle is the angle of the digging blade in the soil and the digging depth is the depth. In recent literature, forward speed has varied from 0.9–7.9km/h, conveyor speed from 0.2–2.37m/s, digger angle from 10–24°, and digging depth from 12–27cm. The full list of publications discussing one of these parameters –over the period under study– and the parameters they used are presented in **Table 2**.

**Table 2:** Harvester specifications in papers from 2017–2022.

Publication	Forward Speed (km/h)	Digging Depth (cm)		Digger Angle (°)	Conveyor Speed (m/s)
Bulgakov et al. (2021)	2.9, 3.6, 5.4, 7.2, 7.9	27		10	1.91
Issa et al. (2020)	2.5, 4.5, 6.5	14–25		12, 17, 22	0.78, 1.11
Hrushetskyi et al. (2021)	7.92	14–25		16–24	–
Bulgakov et al. (2017)	1.9, 2.4, 3.0, 4.0	27		–	1.81–2.37
Sibirev et al. (2019)	3–5.2	12–18		–	1–1.78
Nasr et al. (2018)	1.5, 2.0, 2.5	16, 20, 24		–	–
Hrushetsky et al. (2019)	0.9, 1.8, 2.7, 3.6, 4.5	–		–	–
Gulati (2019)	2.7	–		–	–
Fu et al. (2022)	–	–		–	0.2, 0.4, 0.6, 0.8, 1.0
Poppa et al. (2020)	–	–		–	0.33, 1.00
Wei et al. (2019)	–	–		–	1.54, 1.80, 2.06

Forward speed of the harvester in km/h. Digging depth of the harvester blade in cm. Digger blade angle in °. Conveyor speed in m/s.

- means no data reported.

As much as soil and potato characteristics can help indicate the optimal harvester parameters, the main criterion affecting these parameters is the farmer’s optimization criterion. The farmer has to balance several objectives such as reducing tuber damage and loss while increasing their harvesting efficiency. Varying the forward speed of the harvester can result in a variety of outcomes. One outcome which is impacted by varying the forward speed is tuber damage and loss. For example, Bulgakov et al. (2021) found that increasing forward speed from 2.9–7.9km/h while increasing their rotor diameter from 0.65–1m decreased their tuber damage rate from 4.2% to 1.5%. This is in line with Bulgakov et al. (2017), which shows an increase in forward speed decreases the percentage of damaged tubers greatly, despite the percentage of tubers lost increasing. However, contradictory results have been found by Hrushetsky et al. (2019) and Issa et al. (2020), who found that increasing forward speed increased tuber damage. Additionally, Hrushetsky et al. (2019) witnessed an increase in both tuber loss and damage percentage when increasing forward speed for their design and that of the KST-1,4, which is a standard serial potato digging machine.

Another factor impacted by forward speed is separation efficiency. In Bulgakov et al. (2017); Bulgakov et al. (2021), the impact of forward speed on separation efficiency is studied. In both works, they notice that increasing forward speed up to a point can improve separation efficiency, after which increasing forward speed decreases performance. In Bulgakov et al. (2017), separation efficiency increased slowly up to 2.4km/h after which there was a slow decrease from 2.4 to 3.0km/h. As forward speed is further increased to 4.0km/h a sharp drop in separation efficiency is observed. This is confirmed in Bulgakov et al. (2021), where increasing forward velocity from 2.9–5.4 km/h while increasing the rotor diameter from 0.65–1m improved soil separation. However, when further increasing the forward speed from 5.4–7.9km/h they found that soil separation decreased.

Finally, forward speed also impacts field capacity and harvesting efficiency; Issa et al. (2020) found that in general increasing forward speed, increased actual field capacity and the power required by the harvester, while also decreasing field efficiency and the specific energy consumption of the harvester. Another observation from this paper was that increasing forward speed from 2.5–4.5km/h increased the tuber lifting percentage. Although tuber lifting percentage decreased when further increasing forward speed from 4.5–6.5km/h.

Digging angle and depth are similar as a greater digging angle equates to a greater digging depth. We can reduce tuber loss by varying the digging angle: Issa et al. (2020) found that the lifted potato percentage increased from 87.63% to 95.14% with an increase in digging angle from 12°to 22°. The total potato damage also decreased with an increase in the digging angle. However, increasing the digging angle increased the soil resistance resulting in a decreased actual field capacity and field efficiency alongside an increase in required specific energy and power.

Increasing conveyor speed can also increase tuber damage: Wei et al. (2019) acknowledges that at various stages of the potato-soil separation process, the potato will experience different levels of soil cushioning. As a result, they vary soil-potato proportions, splitting them into three groups: the primary clod-crushing stage (7.83% - 38.55%), intermediate clod-crushing stage (38.55% - 69.28%) and fine clod-crushing stage (59.04% - 69.28%). They also experiment with agitator frequency and amplitude measuring the number of impacts, impact acceleration, impact duration, and velocity change as an indicator of potato bruising and damage probability. Potato bruising was broken into 4 groups: no bruising, slight bruising, moderate bruising, and severe bruising. Varying the potato-soil proportion had a large influence on the harvest quality and the impact characteristics experienced during the separation process. As the potato-soil proportion increased and the soil cushion decreased, the number of impacts and peak impact acceleration increased. A slight increase was seen between the primary and intermediate stages but a significant increase was observed between the intermediate and fine clod-crushing stages. The movement of potatoes on the conveying device also varied depending on the stage. At the primary stage, there was little potato movement, at the intermediate stage the potatoes were rolling, and at the fine stage, potatoes were jumping and rolling increasing the damage probability. As the agitator vibration intensity increased the number of impacts and peak impact acceleration also increased gradually. Consequently, vibration intensity should be selected in order to reduce bruising and mechanical damage while maximizing separation efficiency. The impact of potato-soil proportion was more obvious than that of the conveyor running speed. Although at 2.06m/s, the peak impact acceleration at intermediate and fine potato-soil separation was higher than when the conveyor speed was 1.54 and 1.80m/s. The number of impacts was slightly smaller at 2.06m/s compared to 1.80m/s. They do state that increasing speed, increases separation efficiency, and if the rod-type conveyor speed is too slow it will negatively impact harvesting efficiency. However, increasing the conveyor speed will increase the linear velocity of the potatoes as they fall into the windrows or containers which can cause damage.

An opposing discovery is presented by Bulgakov et al. (2017), who shows that the percentage of soil separation and separation intensity both decrease with an increase in conveyor speed. Finally, Issa et al. (2020) state that the actual field capacity and field efficiency increase with conveyor speed, although they conclude that varying conveyor speed had no significant impact on tuber damage. They also find that an increase in conveyor speed decreased tuber lifting percentage.

### 4.2 Mechanical harvester designs

There is a significant amount of research into the mechanical design of potato harvesters. These designs vary in complexity, from simple designs focused on harvester specifications such as digging depth and forward speed to more complex designs with agitators and rotary components to remove soil clods from the production pipeline.

Designing mechanised potato harvesters has proven to be a constant trade-off between efficiency and potato damage. Designs which improve efficiency while minimizing damage are highly desirable. One common design option which can be altered to optimize this goal is the sub-cultivating working parts of the harvester. These parts are important in breaking up the soil and reducing tuber damage. Done effectively, tuber damage can be reduced and efficiency increased: Hrushetsky et al. (2019) proposed to improve harvesting efficiency with a digging component that utilizes a passive blade with cutting discs and soil compactors. The design reduces the tractive resistance of the potato digger by 18% while improving the buckling rate of the potato-soil layer. Ultimately increasing productivity by 22%, achieving a yield of 13.2 t/ha and a digging completeness of 99.1% compared to the serial digger KST-1,4 which achieved 97.6%. This is similar to the work of Hrushetskyi et al. (2021) who aimed at reducing tuber mechanical damages while providing qualitative indicators of the potato heap separation process. They achieved this by mathematically modelling the movement of particles when the share-board surface of the harvester collided with the potato heap. Similar to the work done by Hrushetsky et al. (2019), they compare their theoretical and experimental results, showing their model to have a deviation within 5%. They concluded that this indicates the adequacy of their mathematical model to simulate the separation process of potato heaps.

A different approach to improving the time efficiency of potato harvesting was taken by Gulati (2019) by designing a two row combine harvester. Their harvester can reduce labor, time, and expenses by harvesting two rows of potatoes at once. The design works again by breaking the soil ridge, exposing the potatoes so they can be easily and efficiently collected. These potatoes are then lifted from the soil and conveyed to the following trolley using a rod-chain separator-conveyor and a swan-neck elevator-conveyor. Two sets of agitators are attached to the conveying system. The purpose of the rod-chain separator-conveyor system with agitators is to remove the soil, stems and debris from the collected potatoes with minimal injuries. Their prototype was able to operate with a single 40 horsepower tractor and has an effective field capacity of 0.26 ha/hr, tuber bruising of 6%, and 98.4% of the excavated potatoes made it to the trolley with a field loss of 1.6%.

**Figure 6:**
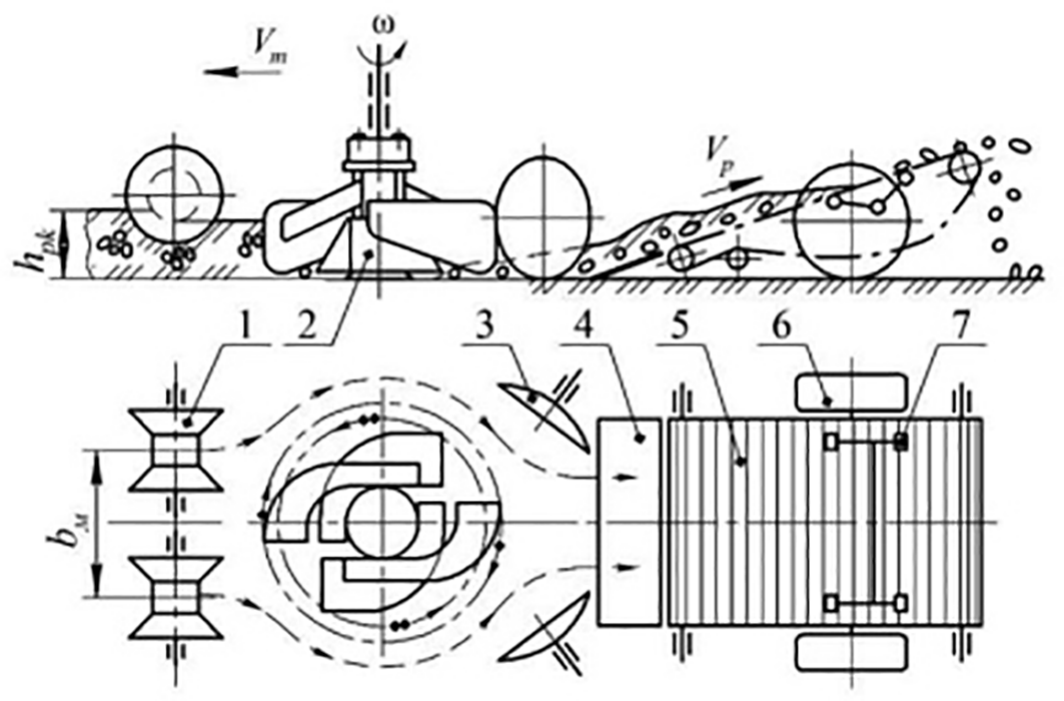
The design of a rotary-type potato harvester to improve soil clod separation. Image is taken from Bulgakov et al. (2021).

Finally, Bulgakov et al. (2017; Bulgakov et al., 2019; Bulgakov et al., 2020; Bulgakov et al., 2021) published four articles during 2017–2021 related to the use of rotary components in potato harvesting. The goal of this research was to clean the potatoes and in particular remove soil clods. This was achieved by a variety of designs however the key connection was that of rotation. Their later publication from 2021, relates to the concept of breaking up the potato-soil layer and therefore will be discussed first. They designed a rotary-type potato harvester that improves soil-clod separation in heavy loam soil [Bulgakov et al. (2021)]. The rotational component was added to help break up the soil, reducing the number of soil clods lifted onto the separation tool. Their proposed design can be seen in **Figure 6**. They varied the translational velocity of the machine, the rotor rotation frequency, the rotor diameter, the rotor circumference and the distance between the spherical discs to determine their effects on performance. They found that the soil separation improves as the rotor diameter increases from 0.65 to 1.0m and translational velocity increases from 0.8 to 1.5m/s. However, when velocity increases from 1.5 to 2.2m/s soil separation decreases. Also, tuber damage rates decrease from 4.2 to 1.5% when rotor diameter increases from 0.65 to 1.0m and translational velocity increases from 0.8 to 2.2m/s. When the distance between the rotors’ circumference and the spherical discs increases, the tuber damage rate also increases. The maximum soil separation reached was 93.5%.

**Figure 7:**
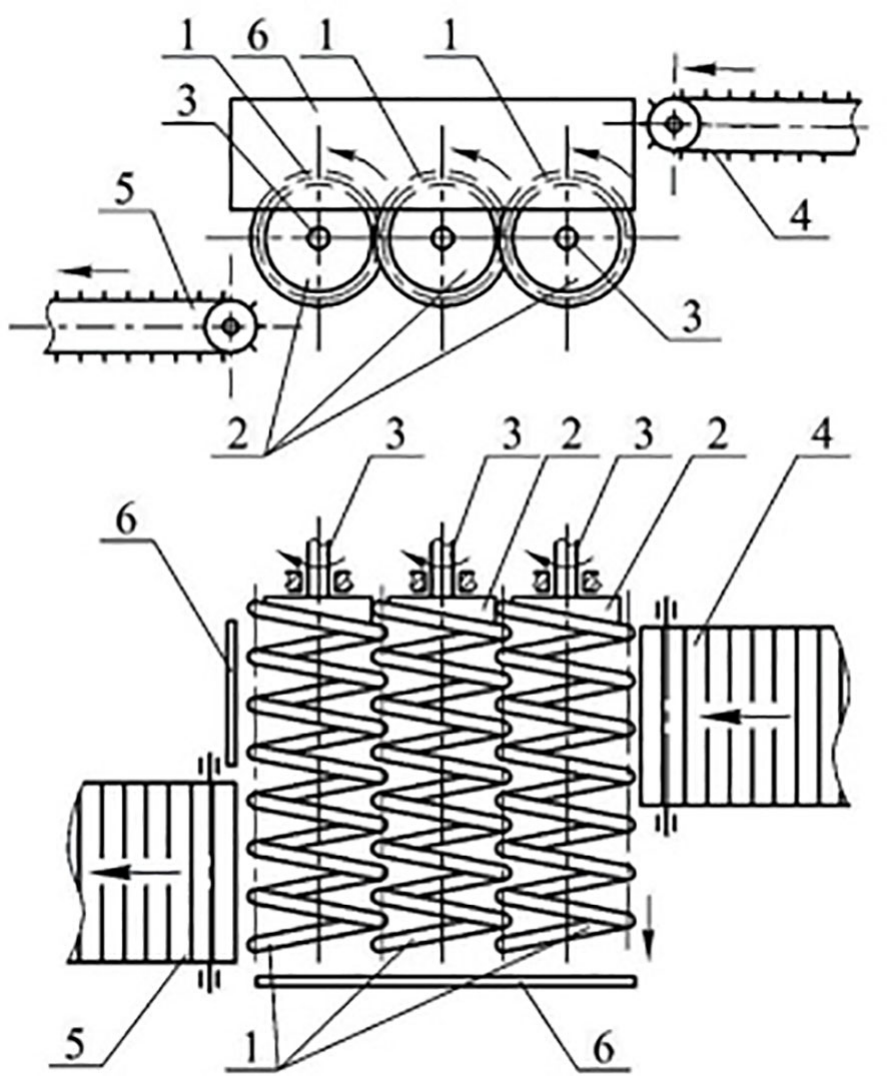
The spiral potato heap separator design. Image taken from Bulgakov et al. (2017).

Other approaches by the same authors, discuss the concept of a spiral soil separator that can be included in the conveyor system. For example, Bulgakov et al. (2017) proposed a novel design for a spiral potato heap separator. This design can be seen in **Figure 7**. They believe that their spiral separator in conjunction with other technical solutions such as agitators can self-clean the rollers resulting in improved soil separation. Their initial experiments corroborated this belief. They found the optimal parameters to be: a peripheral speed of rotation of 1.75–2.0m/s; an inclination angle of the separator to the horizon of 15–19°; and the installation eccentricity of the spirals as 5–10mm. The recommended forward speed was 0.6–0.8m/s (2.16–2.88km/h). Increasing the inclination angle of the separator and eccentricity of the spirals increased soil sifting and separation intensity. Conversely, increasing the peripheral speed of rotation towards 2m/s gradually decreased the percentage of sifted soil. After 2m/s a rapid decrease in the percentage of sifted soil was observed, this is due to a reduction in the contact time between the potato-soil mixture and the separator.

The concept of a spiral separator was further developed in their work, Bulgakov et al. (2019). In this paper, they discuss a theoretical design with the goal of removing soil clods and unwanted debris. They define a mathematical model for sieving potatoes on a spiral separator and use Matlab to compare the impact of different variables on the time taken to remove soil clods. They find that as the angular velocity goes from 10 to 50 rad/s the time to complete sieving goes from 0.07 to 0.025s. As the spiral’s radius goes from 0.1 to 0.3m the time to complete sieving goes from 0.04 to 0.01s. Increasing the cleaning spiral’s helix angle from 10°to 30°at a radius of 0.1m reduces the time to complete sieving from 0.75 to 0.026s, at a radius of 0.28m it goes from 0.28 to 0.005s. Varying the amplitude of oscillation of the spiral does not significantly impact the soil clod’s residual mass.

Later in 2020, Bulgakov et al. (2020) implemented the spiral separator with the goal of removing more clods, soil, plant debris, and stones on the field so it is better environmentally. The spiral potato cleaner contained three cleaning spirals mounted as cantilevers. One end of each spiral is fixed on the hubs connected to the driving shaft. The soil mixture is dropped from a small height, which partially destroys the soil layer around the potato. Since the spirals are cantilevers the free ends make oscillatory movements in the longitudinal-vertical plane. There are gaps between the spirals which allow small soil clods and plant debris to fall through. The theoretical study of the motion and sifting of a body on the surface of the spiral-type potato cleaner is based on the basic principles of the dynamics of the motion of a body of variable mass. Their equation takes into account that the mass of the soil clod will decrease over time. Field experiments were used to determine the performance of the potato cleaner. The following indicators were used to determine the quality of the spiral-type potato cleaner: the screening ability of the cleaner, the intensity separation of admixtures, and the specific separation intensity. They then performed regression on each quality indicator. The cleaning ability of their design can be improved by altering the angular velocity, the initial angle of inclination, and the radius of the spirals. A soil clod reduction of 95% in the time range of 4.8–7.2s was achieved. Similar to conveyor speed, too fast of an angular velocity reduces the contact time between the soil clods and the spirals, reducing the potato cleaner’s separation performance. Decreasing the initial angle of contact between the potato-soil layer and the spiral cleaner positively impacts the separation rate of the soil admixtures from the potato heap.

## 5 Trends in potato harvesting

One trend identified during the review was the use of electronic potatoes to understand the impact forces applied on the potato throughout the harvesting process. This is important not only when designing a potato harvester but also when selecting the harvester specifications. Electronic potatoes are objects designed to be as similar as possible to actual potatoes while containing sensors that can record the forces exerted on them. They have been utilized by Sibirev et al. (2019), to determine the impact forces experienced by potatoes during the full harvesting process for three different potato harvesters: AVR-Spirit-6200, Dewulf RA-3060 and Bolko. This study varied the forward speed, depth of the ploughshare in the soil, and the speed of the open-web elevator to determine their influence. However, the difficulty with electronic potatoes revolves around correctly modelling the potato in order to gain accurate measurements. One paper using the coefficient of restitution and the static modulus of elasticity to better model the impact characteristics and elasticity of potatoes is Surdilovic et al. (2018). The aim of this paper is to better understand the forces applied to potatoes when they fall. They found that all bar one of their dummies did not accurately represent real potatoes. Noting that the dummy potatoes had a higher maximum impact and acceleration with a lower deformation.

Several publications, in the period under study, look to change the status quo of potato harvesting procedures. The first of which is that farmers are currently not accurately reporting the waste generated during potato harvesting. As stated by Schneider et al. (2019), undersized potatoes that get composted should be reported as waste. Subsequently, they provide a practical approach for determining potato losses directly on the field. Their study included two farms, one in Austria, and the other in Germany. They consider two types of loss, type one, those remaining in the soil not collected by the harvester, and type two, those sorted out due to technical or quality reasons. In Austria, they used a net to catch type two, the net also helped to represent the area that needed to be excavated to find type one. In the German farm, the farmer de-haulms the potatoes prior to harvest and plants mustard plants. The roots of the mustard plant loosen the soil and elevate the potatoes. Due to this elevation, the potatoes are easier to extract from the soil which allows the harvester to drive faster. Small potatoes at the root of the plant are not economically viable for farmers to collect. As a result, they set shallower digging angles to save fuel. These smaller potatoes are often automatically filtered out by potato harvesters as they fall through gaps in the conveyor system which are intended to remove soil clods from the system. In Austria, loss two was higher than loss one while in Germany loss one was higher than loss two. The German farm on average produced larger potatoes which were cut in half by the harvester. This in conjunction with several smaller potatoes caused loss one to outnumber loss two. Overall, the loss in Germany was 1.4% compared to 9.1% in Austria. They conclude that losses during primary production are highly variable depending on region, weather, type of crop as well as cultivator and harvest method. They surmise that the harvester specifications such as digging depth and forward speed have a big impact on tuber loss. Their final proposal uses 2-4 people to determine loss, by collecting and weighing the potatoes on the field.

Another trend potentially interrupting the status quo around the world is the push to use more renewable energy. In particular, the trend towards electric vehicles, and potato harvesting is not exempt from such changes: Muneer and Dowell (2022) provides a case study on the use of renewable energy on a potato farm in Scotland, UK. In the case study, they compare the prices of different energy sources. They show that the cost of generating one kWh of energy using solar and wind power is lower than coal, gas, geothermal and nuclear. And that the cost has dropped significantly in the last 10 years as renewable technology improves. In order to prove that renewable energy is appropriate when potato harvesting they need to ensure that power is consistently supplied to the farm year-round and that the equipment used to generate the energy will not need to be replaced frequently. To measure the performance of the wind turbine they measure the average wind speed (m/s), average power (kW), and capacity factor (ratio) for wind turbines across the years of their experiment as well as across the months of 2015. They also provide the energy generated and capacity factor for solar power. Solar power generates the most energy in summer, while wind generates the most energy in the winter. In Scotland wind power generates more energy than solar power. A combination of the two can provide enough energy year-round to harvest and store potatoes. They notice that the months from March to June produce the most energy when combining both sources of energy. They also state that if maintained correctly then the output from both solar and wind energy does not deteriorate significantly in the first eight years. With some countries signing on to meet specific climate targets the pressure placed on agriculture to reduce its emissions will increase. This may lead to more farms following the blueprint provided in this article and therefore electronic tractors and potato harvesters may increase in demand.

Finally, McPhee et al. (2020) attempts to model the impact of low-mass autonomous vehicles on soil bulk density using COMPSOIL. They also look at the critical soil bulk density and what this means for harvesting two different crops, one of which is potato. They determine suitability in terms of operational capacity and what this means logistically for farming operations. They wish to determine the correct size of machine which will reduce traffic-induced soil compaction while still meeting a certain standard of productivity. They determine that a medium-sized autonomous fleet integrated into a Controlled Traffic Farming (CTF) approach would be best equipped to meet these requirements. However, CTF is not suitable for root and tuber farming as the harvester must currently drive over the top of the crops. They also state that even low-mass autonomous vehicles breach critical bulk density and therefore are not a solution for avoiding soil compaction in potato harvesting. They claim that alternative harvester designs must be created to avoid soil compaction for potato harvesting.

## 6 Discussion

A better understanding of potato characteristics can improve the design of the equipment involved in the harvesting and post-harvesting processes. However, publications such as Ahangarnezhad et al. (2019) need to ensure they develop upon previous work in the field so as to not waste time repeating the work of others. This paper for example only discussed one other paper which explores the physical and mechanical properties of the potato. Despite this being a common area of research, especially in the creation of electronic potatoes. They could also help to further develop the community and improve the repeatability of their experiment by providing the soil type and growing conditions for the potato they used in their experiments. The following subsections, are the main outcomes of the analysis covered in this work.

### 6.1 Farming land vs. population

**Figure 8:**
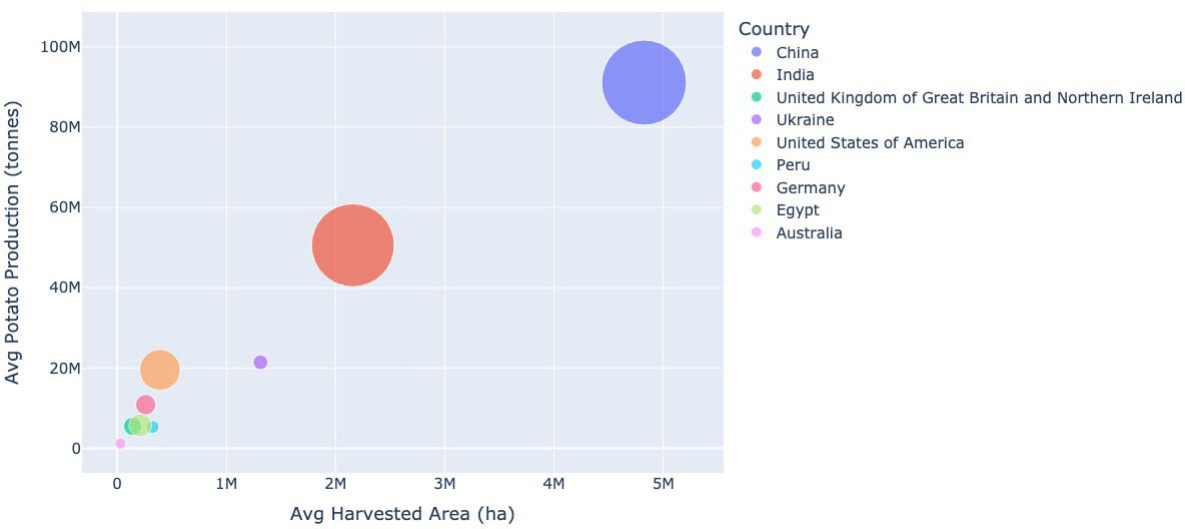
The average potato production (tonnes) between 2017–2021, against the average harvested area dedicated to growing potatoes (ha) for the same time period for each country displayed. The size of each circle equates to the size of that countries population. Data extracted from Dataset FAO (2022a) and Dataset FAO (2022b).

**Figure 9:**
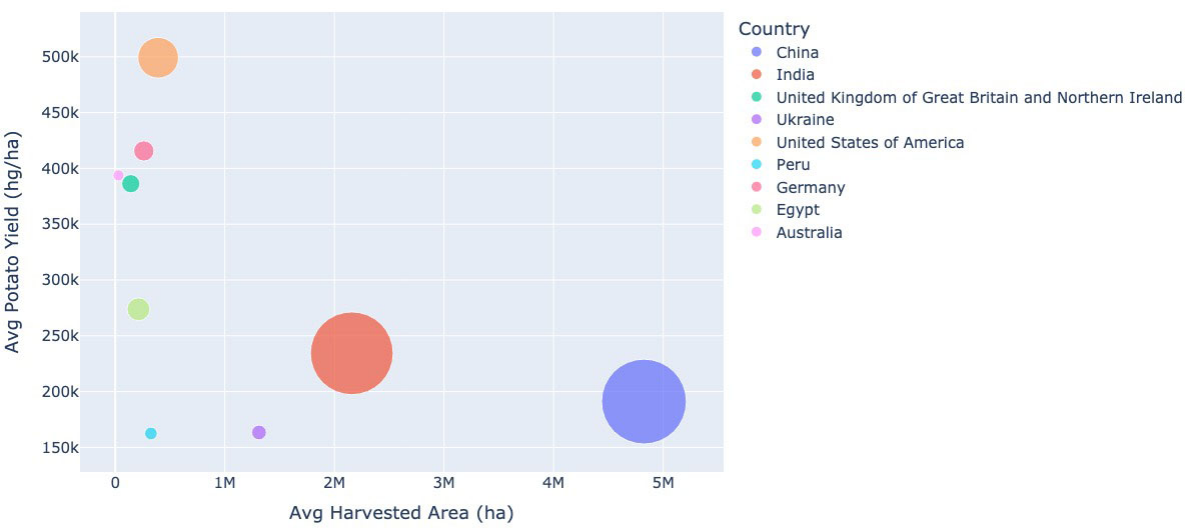
The average yield (hg/ha) between 2017–2021, against the average harvested area dedicated to growing potatoes (ha) for the same time period for each country displayed. The size of each circle equates to the size of that countries population. Data extracted from Dataset FAO (2022a) and Dataset FAO (2022b).

The average population, harvested area, production and yield are used to produce **Figures 8**, **9**. In **Figure 8**, the average potato production for 2017–2021 is plotted against the average harvested area for this time period. This graph shows that generally, the larger the average harvested area the higher the average potato production. The size of each circle equates to the average population of the country. Countries with a larger population tend to produce more potatoes than those with a smaller population.

The opposite relationship between potato production and harvested area is seen when comparing the average potato yield against the average harvested area for 2017–2021 (see **Figure 9**). As the average harvested area increases the average potato yield decreases. Again, the population size is represented by the size of the circle. However in this case there appears to be no clear relationship between the population size and yield.

### 6.2 Conflicting harvester specifications

Harvester specifications are specific to the field and design of the potato harvester. Therefore research can often appear to contradict one another. For example, Bulgakov et al. (2021) states that increasing forward speed decreases tuber damage while Issa et al. (2020) found that increasing forward speed increased damage. There are two important factors to discuss here. Firstly soil type, Bulgakov et al. (2021) performed their experiments in heavy loam soil which is notoriously difficult to harvest in due to the high percentage of soil clods. In Issa et al. (2020), experiments were performed over sandy clay, which is a more preferable environment for potato harvesters. Most well-known potato combine harvesters are built to only operate in sandy soils (see Bulgakov et al. (2021)]. The second is the design of the harvester. The harvester design influences how forces are applied to the soil and potato. Therefore changing the harvesting specifications will vary their impact. Different designs will have different optimal harvester specifications.

### 6.3 Levels of automation

This section looks at the current level of automation present in each of the countries discussed in this review. The levels of automation described here are based loosely on those presented by the SAE International On-Road Automated Driving Committee, O (2021). The levels however differ slightly and their definitions are presented below: Level 0 equates to hand harvesting. Level 1 is a semi-mechanised harvester. Level 2 is a fully-mechanised harvester. Level 3 is partial automation of the harvesting process. Level 4 is the full automation of the harvesting process. Level 5 is the full automation of the potato farming process.

The only work reporting on automated potato harvesting was McPhee et al. (2020). However it was a hypothetical proposal, no potato harvester was actually automated. As such the highest level of automation was achieved by Fu et al. (2022), with their autonomous potato cleaner. This device was not attached to a harvester and therefore it is not considered part of the harvesting process. Since no other paper discussed automation, the top level of automation in potato harvesting is therefore Level 2. There were no potato harvesting papers produced by Peru and therefore it was not assigned a level of automation. However, based on surrounding countries, it is likely that Peru is Level 1. **Table 3** summarizes the automation levels of potato harvesting in the different countries under study (over the period covered in this review).

**Table 3:** The levels of potato harvesting automation, number of potato harvesting based journal publications between 2017–2022; as well as production and yield in 2021 for the top potato producing countries by continent.

Countries	Automation Level	Number of Publications	Potato Production (tonnes)	Yield (hg/ha)
China	2	5	**94,362,175.0**	163,179.0
Ukraine	2	**6**	21,356,320.0	166,430.0
India	2	1	54,230,000.0	241,237.0
Germany	2	3	11,312,100.0	437,944.0
UK	2	1	5,306,719.8	387,352.0
Australia	2	1	1,267,638.6	403,372.0
USA	2	1	18,582,370.0	**490,727.0**
Egypt	1	2	6,902,817.0	262,758.0
Peru	–	0	5,661,443.0	171,245.0

Bold values means larger value.

China, India, Germany, and Australia were all assigned Level 2 due to reviewed papers from these countries discussing fully-mechanised harvesters [Fu et al. (2022); Gulati (2019); Schneider et al. (2019); McPhee et al. (2020)]. Ukraine, the USA, and the UK were also assigned Level 2, though this decision was arrived at based on additional papers not included in the survey [Bulgakov et al. (2022); Spang and Stevens (2018); Godwin et al. (1999)]. The UK and Germany are also part of NWEC-05 which as discussed by Goffart et al. (2022) has a very high level of mechanization, this confirmed their assignment as Level 2. Egypt was assigned Level 1 based on their reviewed papers [Nasr et al. (2018; Nasr et al., 2019)] proposing a semi-mechanised potato harvester.

The following arguments can be made to change the automation level for China, India, and Ukraine. China and India are both primarily semi-mechanised harvesting countries [Wei et al. (2019); Gulati (2019)]. Despite this, they have both produced papers in the last five years discussing the use of fully-mechanised harvesters [Gulati (2019); Fu et al. (2022)]. As a result, both have been assigned an automation Level 2.

According to Hrushetskyi et al. (2021) and Hrushetsky et al. (2019) the majority of Ukrainian potato harvesting is carried out manually, despite previously most harvesting being mechanised. The majority of potato harvesters are imported from Russia, Belarus and Germany and are outdated. Nevertheless, since Ukrainian research papers discuss fully-mechanised approaches [Bulgakov et al. (2022)] they have been assigned an automation Level 2.

## 7 Conclusion and future work

Potato harvesting is a complex problem as the optimal solution varies around the world. Potato and soil characteristics contribute to the selection of an optimal harvesting technique and harvester specification. In the last five years, automation in potato harvesting has been discussed hypothetically but not implemented. Subsequently, the highest level of automation is fully mechanised harvesting (Automation Level 2). In recent literature, the design of mechanical potato harvesters has revolved around the breaking up and removal of soil clods. In addition to an improved ability to remove soil clods, future harvesters may also be electric as the need to reduce the environmental impact of farming increases. Intelligent systems such as electronic potatoes can help to reduce tuber damage and loss by understanding the forces exerted on the potato during harvesting. Nevertheless, there is a gap for intelligent systems in potato harvesting research. Introducing these intelligent systems may help to ease the strain placed on the agricultural sector caused by a shrinking workforce and an increasing population.

## Author contributions

FA contributed to the conception and design of the study. CJ organized the papers and data, performed the analysis, and wrote the first draft. All authors contributed to the article and approved the submitted version.

## Appendix A1

**APPENDIX TABLE 1:** China’s potato production (in tonnes), yield (in hg/ha), area harvested (in ha), and population for the years 2017–2021.

Year	Production	Yield	Area Harvested	Population
2017	88,536,429.0	182,085.0	4,862,361.0	1,442,041,109
2018	90,321,442.0	189,722.0	4,760,724.0	1,448,928,199
2019	89,562,447.0	221,750.0	4,038,885.0	1,453,801,543
2020	92,852,722.1	198,588.0	4,675,654.0	1,456,928,486
2021	**94,362,175.0**	163,179.0	**5,782,738.0**	**1,457,934,562**
Mean	**91,127,043.0**	191,064.8	**4,824,072.4**	**1,451,926,779.8**
Std.	2,411,031.0	21,553.6	625,113.2	6,544,906.9
% Change	6.6	-10.4	18.9	1.1

The mean and standard deviation for each column across the four years is provided, as well as the percentage change between the years 2017 and 2021. Bold text indicates that is the largest value across all countries in the study, except standard deviation where it is the smallest value that is bold. Data extracted from Dataset FAO (2022a) and Dataset FAO (2022b).

## Appendix A2

**APPENDIX TABLE 2:** India’s potato production (in tonnes), yield (in hg/ha), area harvested (in ha), and population for the years 2017–2021.

Year	Production	Yield	Area Harvested	Population
2017	48,605,000.0	223,061.0	2,179,000.0	1,354,195,680
2018	51,310,000.0	239,542.0	2,142,000.0	1,369,003,306
2019	50,190,000.0	230,971.0	2,173,000.0	1,383,112,050
2020	48,562,000.0	236,772.0	2,051,000.0	1,396,387,127
2021	54,230,000.0	241,237.0	2,248,000.0	1,407,563,842
Mean	50,579,400.0	234,316.6	2,158,600.0	1,382,052,401.0
Std.	2,344,165.5	7,401.1	71,535.3	21,235,092.6
% Change	11.6	8.2	3.2	3.9

The mean and standard deviation for each column across the four years is provided, as well as the percentage change between the years 2017 and 2021. Bold text indicates that is the largest value across all countries in the study, except standard deviation where it is the smallest value that is bold. Data extracted from Dataset FAO (2022a) and Dataset FAO (2022b).

## Appendix A3

**APPENDIX TABLE 3:** Germany’s potato production (in tonnes), yield (in hg/ha), area harvested (in ha), and population for the years 2017–2021.

Year	Production	Yield	Area Harvested	Population
2017	11,720,000.0	467,864.0	250,500.0	82,624,374
2018	8,920,800.0	353,719.0	252,200.0	82,896,696
2019	10,602,200.0	390,361.0	271,600.0	83,148,141
2020	11,715,100.0	428,340.0	273,500.0	83,328,988
2021	11,312,100.0	437,944.0	258,300.0	83,408,554
Mean	10,854,040.0	415,645.6	261,220.0	83,081,350.6
Std.	1,172,814.0	44,326.5	10,762.8	**322,402.0**
% Change	-3.5	-6.4	3.1	1.0

The mean and standard deviation for each column across the four years is provided, as well as the percentage change between the years 2017 and 2021. Bold text indicates that is the largest value across all countries in the study, except standard deviation where it is the smallest value that is bold. Data extracted from Dataset FAO (2022a) and Dataset FAO (2022b).

## Appendix A4

**APPENDIX TABLE 4:** UK’s potato production (in tonnes), yield (in hg/ha), area harvested (in ha), and population for the years 2017–2021.

Year	Production	Yield	Area Harvested	Population
2017	6,218,000.0	425,890.0	146,000.0	66,064,804
2018	5,060,000.0	361,429.0	140,000.0	66,432,993
2019	5,307,000.0	368,542.0	144,000.0	66,778,659
2020	5,512,813.1	388,226.0	142,000.0	67,059,474
2021	5,306,719.8	387,352.0	137,000.0	67,281,039
Mean	5,480,906.6	386,287.8	141,800.0	66,723,393.8
Std.	442,174.1	25,030.5	3,492.9	486,067.2
% Change	-14.7	-9.1	-6.2	1.8

The mean and standard deviation for each column across the four years is provided, as well as the percentage change between the years 2017 and 2021. Bold text indicates that is the largest value across all countries in the study, except standard deviation where it is the smallest value that is bold. Data extracted from Dataset FAO (2022a) and Dataset FAO (2022b).

## Appendix A5

**APPENDIX TABLE 5:** Ukraine’s potato production (in tonnes), yield (in hg/ha), area harvested (in ha), and population for the years 2017–2021.

Year	Production	Yield	Area Harvested	Population
2017	22,208,220.0	167,837.0	1,323,200.0	44,657,257
2018	22,503,970.0	170,498.0	1,319,900.0	44,446,954
2019	20,269,190.0	154,869.0	1,308,800.0	44,211,094
2020	20,837,990.0	157,244.0	1,325,200.0	43,909,666
2021	21,356,320.0	166,430.0	1,283,200.0	43,531,422
Mean	21,435,138.0	163,375.6	1,312,060.0	44,151,278.6
Std.	930,361.5	6,890.6	17,333.2	444,301.4
% Change	-3.8	-0.8	-3.0	-2.5

The mean and standard deviation for each column across the four years is provided, as well as the percentage change between the years 2017 and 2021. Bold text indicates that is the largest value across all countries in the study, except standard deviation where it is the smallest value that is bold. Data extracted from Dataset FAO (2022a) and Dataset FAO (2022b).

## Appendix A6

**APPENDIX TABLE 6:** USA’s potato production (in tonnes), yield (in hg/ha), area harvested (in ha), and population for the years 2017–2021.

Year	Production	Yield	Area Harvested	Population
2017	20,453,430.0	483,887.0	422,690.0	329,791,231
2018	20,421,560.0	497,274.0	410,670.0	332,140,037
2019	19,251,320.0	507,522.0	379,320.0	334,319,671
2020	19,051,790.0	**516,365.0**	368,960.0	335,942,003
2021	18,582,370.0	490,727.0	378,670.0	336,997,624
Mean	19,552,094.0	**499,155.0**	392,062.0	333,838,113.2
Std.	844,024.8	12,979.5	23,236.5	2,911,248.8
% Change	-9.2	1.4	-10.4	2.2

The mean and standard deviation for each column across the four years is provided, as well as the percentage change between the years 2017 and 2021. Bold text indicates that is the largest value across all countries in the study, except standard deviation where it is the smallest value that is bold. Data extracted from Dataset FAO (2022a) and Dataset FAO (2022b).

## Appendix A7

**APPENDIX TABLE 7:** Peru’s potato production (in tonnes), yield (in hg/ha), area harvested (in ha), and population for the years 2017–2021.

Year	Production	Yield	Area Harvested	Population
2017	4,776,294.0	153,875.0	310,400.0	31,605,486
2018	5,133,927.3	159,012.0	322,864.0	32,203,944
2019	5,389,231.0	162,730.0	331,177.0	32,824,861
2020	5,515,378.0	165,551.0	333,153.0	33,304,756
2021	5,661,443.0	171,245.0	330,604.0	33,715,471
Mean	5,295,254.7	162,482.6	325,639.6	32,730,903.6
Std.	348,828.9	**6,564.9**	9,377.0	844,357.6
% Change	18.5	**11.3**	6.5	6.7

The mean and standard deviation for each column across the four years is provided, as well as the percentage change between the years 2017 and 2021. Bold text indicates that is the largest value across all countries in the study, except standard deviation where it is the smallest value that is bold. Data extracted from Dataset FAO (2022a) and Dataset FAO (2022b).

## Appendix A8

**APPENDIX TABLE 8:** Egypt’s potato production (in tonnes), yield (in hg/ha), area harvested (in ha), and population for the years 2017–2021.

Year	Production	Yield	Area Harvested	Population
2017	4,841,040.0	277,724.0	174,311.0	101,789,386
2018	4,960,062.0	289,282.0	171,461.0	103,740,765
2019	5,200,563.0	292,876.0	177,569.0	105,618,671
2020	6,786,340.0	246,203.0	275,640.0	107,465,134
2021	6,902,817.0	262,758.0	262,706.0	109,262,178
Mean	5,738,164.4	273,768.6	212,337.4	105,575,226.8
Std.	1,019,114.6	19,381.0	52,129.5	2,952,333.2
% Change	**42.6**	-5.4	**50.7**	**7.3**

The mean and standard deviation for each column across the four years is provided, as well as the percentage change between the years 2017 and 2021. Bold text indicates that is the largest value across all countries in the study, except standard deviation where it is the smallest value that is bold. Data extracted from Dataset FAO (2022a) and Dataset FAO (2022b).

## Appendix A9

**APPENDIX TABLE 9:** Australia’s potato production (in tonnes), yield (in hg/ha), area harvested (in ha), and population for the years 2017–2021.

Year	Production	Yield	Area Harvested	Population
2017	1,105,194.2	389,534.0	28,372.0	24,590,334
2018	1,188,655.0	399,682.0	29,740.0	24,979,230
2019	1,225,273.6	378,022.0	32,413.0	25,357,170
2020	1,076,780.1	397,971.0	27,057.0	25,670,051
2021	1,267,638.6	403,372.0	31,426.0	25,921,089
Mean	1,172,708.3	393,716.2	29,801.6	25,303,574.8
Std.	**80,295.6**	10,133.2	**2,181.7**	532,074.5
% Change	14.7	3.6	10.8	5.4

The mean and standard deviation for each column across the four years is provided, as well as the percentage change between the years 2017 and 2021. Bold text indicates that is the largest value across all countries in the study, except standard deviation where it is the smallest value that is bold. Data extracted from Dataset FAO (2022a) and Dataset FAO (2022b).
